# A New Route for Indirect Mineralization of Carbon Dioxide–Sodium Oxalate as a Detergent Builder

**DOI:** 10.1038/s41598-019-49127-8

**Published:** 2019-09-06

**Authors:** Chen Li, Lijie Wang, Min Yuan, Hong Xu, Jinxiang Dong

**Affiliations:** Research Institute of Special Chemicals, College of Chemistry and Chemical Engineering, Taiyuan University of Technology, Taiyuan, 030024 Shanxi P.R. China

**Keywords:** Environmental chemistry, Chemical engineering

## Abstract

Here, we bridge the gap between carbon mineralization and laundry detergent builder with sodium oxalate. Daily laundry can help mineralize carbon dioxide. First, we screen an environment-friendly process to produce sodium oxalate, using CO_2_ as a raw material. Then, we evaluate the properties of sodium oxalate as a detergent builder and prove the formation of calcium oxalate under laundry conditions. Our data suggest that sodium oxalate has excellent calcium-removing properties. Detergents based on sodium oxalate have good detergency. Furthermore, solid calcium oxalates (calcium oxalate monohydrate or calcium oxalate dihydrate, which has good stability in water and thermal stability), is obtained from washing waters. These results demonstrate the possibility of using sodium oxalate as detergent builder. The whole process can transform the greenhouse gas CO_2_ into commodity chemicals and can mineralize carbon.

## Introduction

The emission of greenhouse gases, among which CO_2_ contributes over 60%, is the leading cause of global warming^1^. Fossil fuels for the production of electricity account for roughly a quarter of all CO_2_ emissions^2^. It is impossible to sharply decrease the number of fossil-fuel power plants used to meet the requirement for electricity over a short period^3,4^. Thus, capture and utilization of carbon dioxide from fossil-fuel-burning power plants becomes particularly important.

Physical adsorption, membrane separation, cryogenic separation, and chemical absorption are the most commonly applied methods of capturing CO_2_^5–7^. Each method has its disadvantages. The physical adsorption methods are significantly affected by temperature and pressure, low CO_2_ partial pressure will cause low capture efficiency^8^. At separation conditions, membrane separation is difficult to achieve high purity CO_2_ and high permeability at the same time^9^. The cryogenic methods are applicable only to high CO_2_ concentrations in gas streams and require significant amounts of energy^10^. Chemical absorption is widely recognized as a practical method for industrial-scale development because of its commercial material and the low-partial-pressure applicability of CO_2_ in flue gas^5^. Unfortunately, this method suffers from energy consumption and adsorbent degradation during regeneration, which makes it energy-inefficient^11–13^.

According to the previous publication, we screen an oxalate manufacturing method using CO_2_ as a feedstock^14^. The manufacturing method for oxalates designed is divided to three steps: first, hydroxide was used to adsorb CO_2_ and formed bicarbonate; second, the bicarbonate hydrogenation to formate; finally, the formate is heated to a temperature of 320~440 °C and forms oxalate. The first step and last step are the mature technology^15,16^ and the second step has been extensively researched^17–20^. (The details of the process is shown in the Fig. S1). This method avoids the massive energy consumption of the absorbent regeneration process^21,22^. However, oxalates have limited industrial demand. To expand the application of oxalate, we investigated the possibility of using oxalate as a detergent builder. The early literature mentions that oxalate can act as a non-phosphate detergent builder^23,24^. However, there are no detailed experimental data on the building action of oxalate with different surfactants, and there are no comparisons with other common builders. There is no report about the industrial application of oxalate as a laundry detergent builder so far. Sodium salt is the cheapest among the soluble oxalates; hence, we focus on sodium oxalate.

Sodium oxalate ionizes to Na^+^ and C_2_O_4_^2−^ in water. Oxalate can combine with hardness ions and form insoluble oxalate salts such as whewellite (CaC_2_O_4_·H_2_O), weddellite (CaC_2_O_4_·2H_2_O). These oxalate salts have low solubility, can endure physical or chemical degradation, and are stable for long periods^25–27^. If sodium oxalate can be used as a detergent builder on a large scale, then quantities of carbon dioxide can be used to produce sodium oxalate. Sodium oxalate binds with hardness ions in water and transforms into calcium oxalate through the laundry process of thousands of households. Finally, we can store carbon dioxide in crystalline calcium oxalate monohydrate (COM, CaC_2_O_4_·H_2_O, whewellite) or calcium oxalate dihydrate (COD, CaC_2_O_4_·2H_2_O, weddellite), which are one of the well-known biominerals in nature^28^.

In order to understand the feasibility of using sodium oxalate as a detergent builder and carbon mineralization in practice, we first investigate the calcium-removing properties of sodium oxalate as a detergent builder. The washing performance of detergents based on sodium oxalate under different conditions was then studied. Finally, the solids collected from different experiments were extensively studied by scanning electron microscopy (SEM), X-ray diffraction (XRD), and thermogravimetry (TG) to ensure the formation of calcium oxalates and its stability. Our results show that sodium oxalate has excellent builder properties, high detergency, and that calcium oxalate obtained after washing is stable and thus can meet the needs of carbon mineralization storage.

## Results

### Calcium-removing properties

Surfactants and builders are the most important ingredients in laundry products. Surfactant efficiency is greatly reduced in hard water, so detergent builder is usually used in conjunction with surfactants, removing Ca^2+^ existing in hard water^29–33^. In order to evaluate the basic properties of sodium oxalate as a detergent builder, the calcium-removing capacity (CRC) and calcium-removing rate (CRR) were measured under different test conditions. Meanwhile, we chose sodium tripolyphosphate (STPP), a traditional phosphate builder, and zeolite 4A (sodium aluminum silicate, a widely used phosphate-free builder) as reference builders.

**Figure 1:**
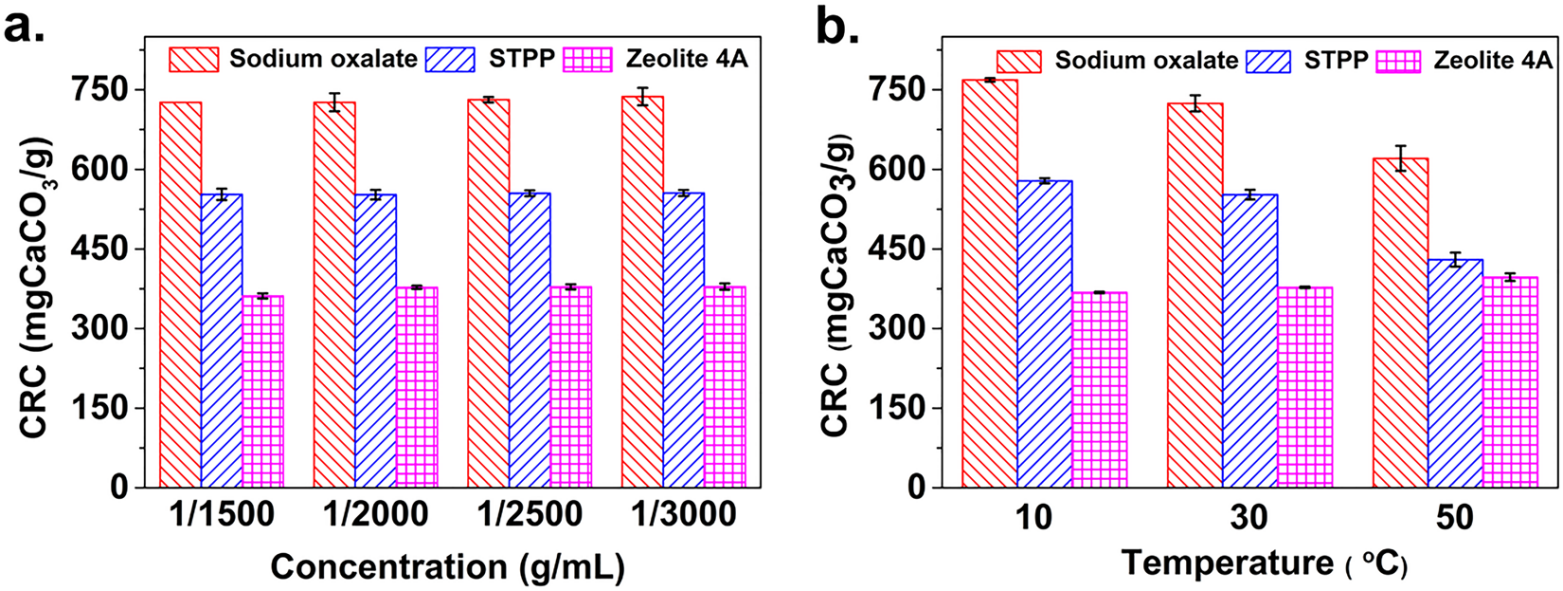
The calcium removing capacity (CRC) of the three builders. (**a**) Different concentrations of the builder under 30 °C. (**b**) Different temperatures under the concentration of the builder at 1/2000. Error bars represent standard deviation.

The CRC of the three builders was determined at concentrations ranging from 1/1500 to 1/3000 g/mL at 30 °C. As shown in Fig. 1a, sodium oxalate showed the best CRC among the three builders. As the builder concentration decreases from 1/1500 to 1/3000 g/mL, the CRC values for sodium oxalate, STPP, and zeolite 4A respectively increase from 726 to 737 mg CaCO_3_/g (per g of the builder), from 552 to 555 mg CaCO_3_/g, and from 361 to 379 mg CaCO_3_/g.

The CRC values of the three builders at a concentration of 1/2000 g/mL in solution at 10, 30, and 50 °C are shown in Fig. 1b. The three builders presented different CRC behavior with the temperature change. For sodium oxalate and STPP, the CRC values decreases as the temperature increases; while for zeolite 4A, the CRC value increases as the temperature increases. This is due to the different calcium-removing mechanisms^34,35^. Even as the temperature increases to 50 °C, the CRC of sodium oxalate is 620 mg CaCO_3_/g, which keeps the highest CRC of the three builders.

**Figure 2:**
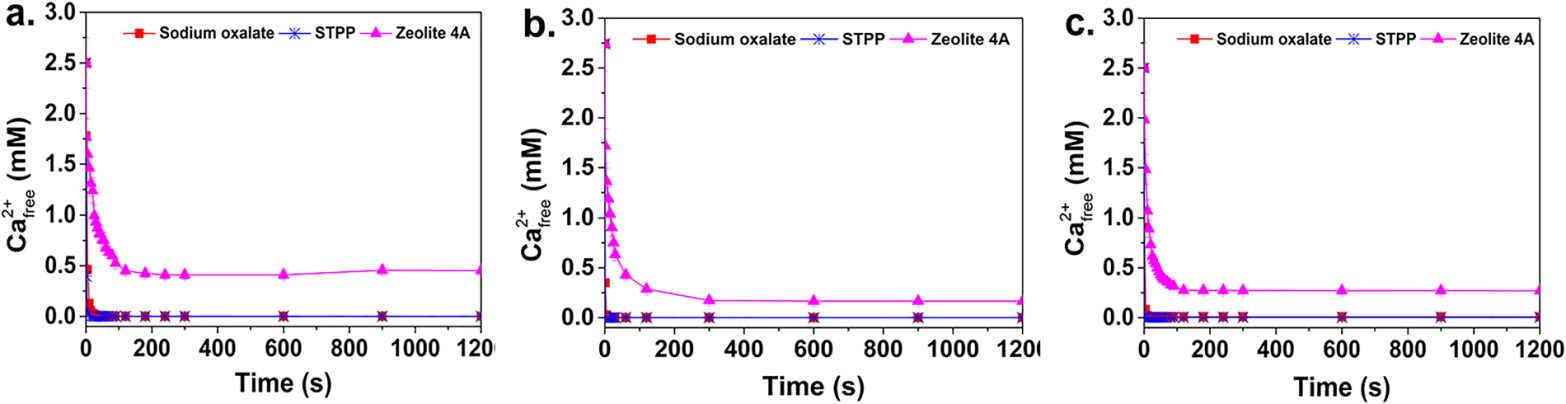
The calcium removing rate (CRR) of the three builders. (**a**) 10 °C; (**b**) 30 °C; (**c**) 50 °C.

The CRR is a measure of the efficiency and depth of calcium-removing property. We monitored the change in free Ca^2+^ concentration in the solution by using a Ca ion selective electrode (ISE), and the testing time was 20 min. The CRR curves of the three builders at different temperatures are shown in Fig. 2. It can be seen that both sodium oxalate and STPP can reduce the free Ca^2+^ concentration to below 10^−5^ mol/L in 1.0 s. Evidently, the calcium-removing efficiency of sodium oxalate and STPP are much better than that of zeolite 4A under the same test conditions. In general, when the free Ca^2+^ concentration is below 10^−5^ mol/L, surfactants can provide good detergency^36^. These results show that sodium oxalate has good builder ability in terms of the CRC and CRR.

### Detergency performance evaluation

Detergency is the most basic property of a detergent. Therefore, sodium oxalate was used in the formulations of detergent according to the usual formulations of detergent powders (the formulations are listed in Table S1 and normal detergent in Table S2)^37^. Anionic surfactants dominate laundry detergent formulations because of their ready supply, low cost, and excellent performance. In this part of the study, we selected three anionic surfactants, sodium dodecyl benzene sulfonate (SDBS, a low-cost and most widely used anion surfactant), methyl ester sulfonate (MES, a new-generation green anion surfactant), and alcohol ether sulfate (AES, a well-performing anionic surfactant). Nonionic surfactants are excellent cosurfactants with anionics^38,39^. We chose alcohol ethoxylated with 9-ethoxy (AEO_9_, an extensively used nonionic surfactant) in this detergent formulation. STPP and 4A were also chosen as reference detergency builders. The selection of other components was based on the usual formulations of detergent powders. All components were first mixed well, and then the detergency was evaluated by using three types of artificial fabrics with different stains: carbon black (JB-01), mixture protein (JB-02), and artificial sebum (JB-03).

**Figure 3:**
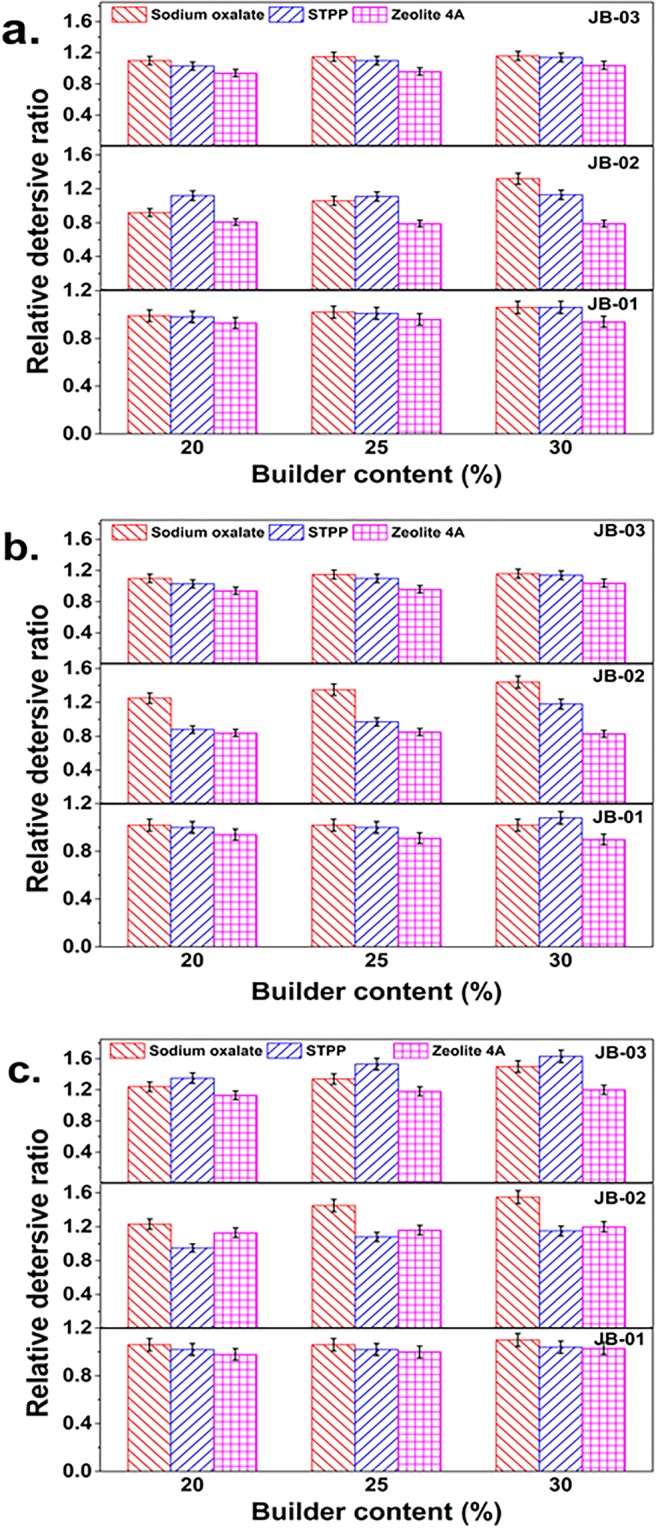
The relative detersive ratio of the three builders with anionic surfactants at 30 °C for 20 minutes. (**a**) SDBS; (**b**) MES; (**c**) AES.

The detergency analysis of relative detersive ratio values is graphically depicted in Fig. 3 (for detailed data, see Table S3). After 27 comparative detergency examinations, we found that the detergency of sodium oxalate is better than that of zeolite 4A and close to that of STPP. It is worth mentioning that detergent formulations based on sodium oxalate have better detersive efficiency with mixture protein soil than those of zeolite 4A and STPP. For example, at a detergency builder content of 30%, the relative detersive ratios of detergent based on sodium oxalate are 1.32 (anionic surfactant SDBS), 1.44 (anionic surfactant MES), and 1.55 (anionic surfactant AES). The relative detersive ratios values for STPP are 1.13, 1.18, and 1.15, respectively; and those for zeolite 4A, are 0.79, 0.83, and 1.03, respectively.

The effect of detergency of sodium oxalate conjunction with anionic surfactants is ranked as follows AES > MES > SDBS.

To further understand the detergency of detergents based on sodium oxalate, tests were also carried out at different washing times and temperatures (Tables S4 and S5). As the wash time is lengthened, the detergency is greatly improved. The reason may be the added mechanical power and adsorption of the surfactant molecules. When the washing temperature decreases from 50 °C to 10 °C, the sodium oxalate based detergent formulations still exhibit a satisfactory detergency; this result suggests that sodium oxalate retains good builder performance in a wide range of washing temperatures. Thus, sodium oxalate as a detergent builder can meet the needs of different washing habits^40,41^.

### Characterization of the solid phase

It is commonly known that solid calcium oxalate has low solubility, and they can be stable for a long time in nature^25–27,42^. Whether calcium oxalate can be obtained under laundry conditions is the most important problem for this research.

In this work, the solid samples were carefully collected from washing water solutions with the oxalate-based detergent formulation at 10, 30, and 50 °C (the formulations are listed in Table S6) and characterized using SEM and XRD. As shown in Fig. S2 and Table 1, we found that calcium oxalates can form at different laundry temperatures. Low washing temperatures tend to form COD (calcium oxalate dihydrate), while high temperatures tend to form COM (calcium oxalate monohydrate).

**Table 1:** The crystal phases of solid collected from washing water solutions with sodium oxalate-based detergent formulations at different temperatures.

	SDBS	MES	AES
10 °C	COD	COD	COD
30 °C	COD	COD	COD
50 °C	COM + COD	COM	COM + COD

**Figure 4:**
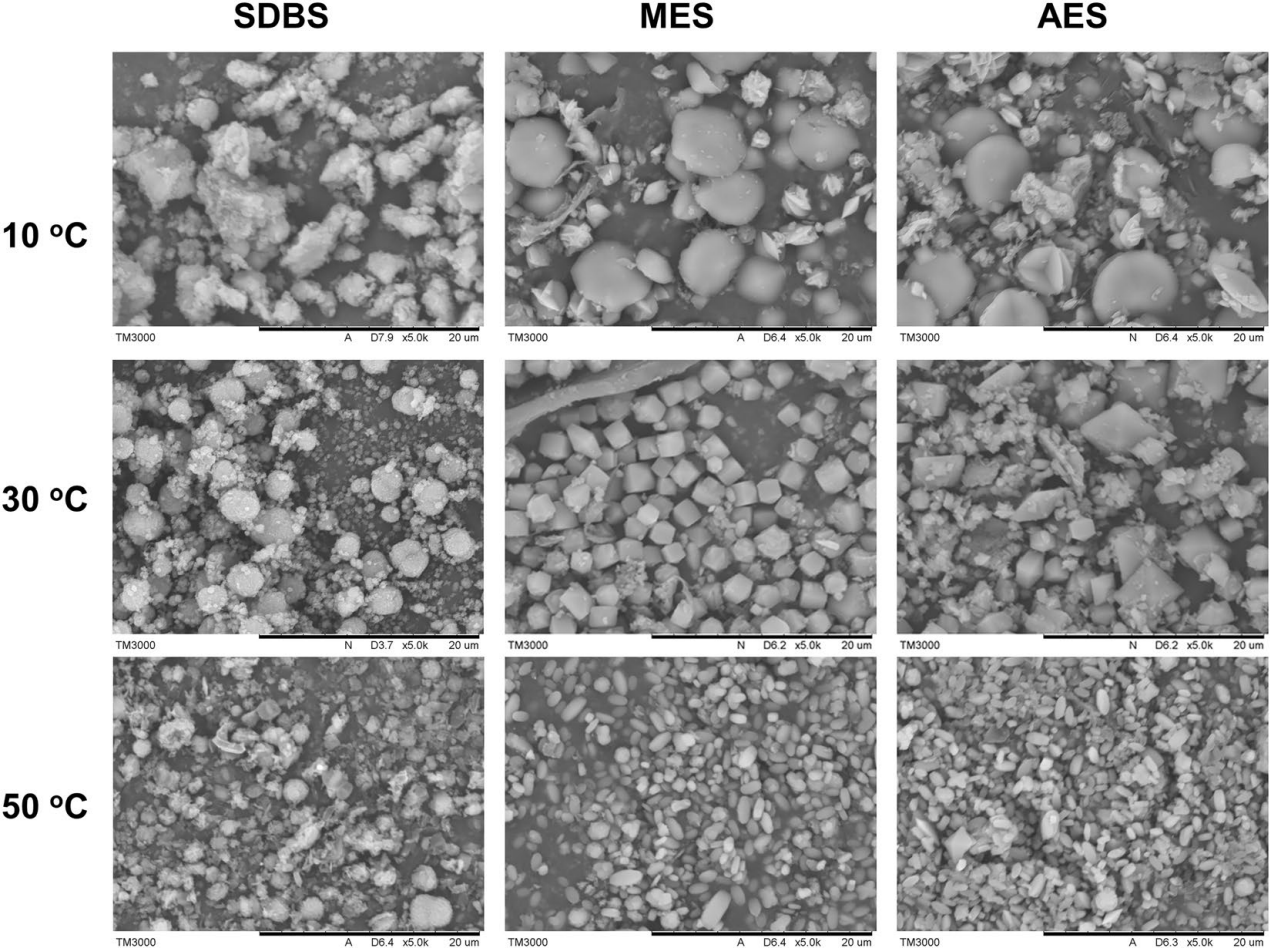
SEM images of solids collected from washing water solutions with sodium oxalate-based detergent formulations at different temperatures.

These changes are also reflected in the morphology. From the SEM images in Fig. 4, the extreme difference in morphologies among the three washing temperatures is visible and high washing temperature tends to form small crystals. At 10 °C, the morphology of the solids obtained from the formulation based on the anionic surfactant SDBS consists of irregular aggregates; solid crystals from MES- and AES-based formulations have similar disc morphologies. As the temperature increased to 30 °C, the crystal morphology of the SDBS-based formulation showed a spherical morphology, while MES was a thick bipyramid and AES was thin a bipyramid. At 50 °C, the crystal particles of the SDBS-based formulation show spherical and fusiform morphologies for MES and AES.

To explore further the regulation of the phase distribution of the obtained solid samples, we also characterized solid samples collected from CRC experiments used only sodium oxalate under laundry conditions. The diffraction patterns of the solid samples obtained from these experiments appear to be the COM phase (JCPDS No. 20-0231; see Fig. S3); the crystals show a typical COM morphology of elongated hexagonal plates (see Fig. S4). Under the washing condition without soiled fabrics, crystalline calcium oxalates were obtained. The detailed phase distribution and morphologies are shown in Figs S5 and S6.

According to the above results, all obtained solid samples contain calcium oxalates, although these solids have different phases or shapes. We found that the morphology of the crystals has a distinctive change at different formulations and temperatures. The results coincide with the fact that surfactants can influence the phase and morphology of calcium oxalate^43–46^. Moreover, the soiled fabrics have a large influence on the phases and morphology of the calcium oxalate, probably because of the influence of stain composition or fabric absorption.

Carbon mineralization storage requires that the mineral materials have good stability. The above results demonstrate that calcium oxalates can be obtained through washing with sodium oxalate based detergent formulations. Next, we studied the thermal and water stability of calcium oxalate. The samples used to measure thermal stability were from laundry conditions without soiled fabrics and using detergents based on sodium oxalate. The TG data for calcium oxalates show that calcium oxalates start to decompose carbon dioxide at a temperature of ~650 °C (Fig. S7). Because of the complex composition and small quantity of solids collected from wash waters, we studied the water stability of calcium oxalates by washing the pure COM and COD using municipal water. COM and COD had no phase change (Fig. S8), and the solid retention rates of COM and COD reached 98.06 and 92.76 wt.%, respectively. The calcium oxalates (either COM or COD) had a good thermal and water stability sufficient to meet the mineralization requirement.

## Discussion

Various technologies have been proposed in order to reduce CO_2_ accumulation in the atmosphere, such as CO_2_ capture, utilization, and storage (storage in geologic formations or minerals). Among these technologies, carbon mineralization is considered an effective way of reducing CO_2_ emissions because of its safety and permanence.

Carbon mineralization storage can be broadly divided into two categories: *in situ* and *ex situ*^47–49^. In *in situ* mineralization, the injected CO_2_ reacts with alkaline rock present in the chosen reservoirs to form solid carbonate species^13^. The high transportation cost to a certain environmental legacy and slow reaction rates have limited its application^50,51^. In *ex situ* mineralization, the carbonates are formed in a separate reactor or industrial process. However, the high energy requirement and costs make *ex situ* mineralization unfeasible^52,53^.

**Figure 5:**
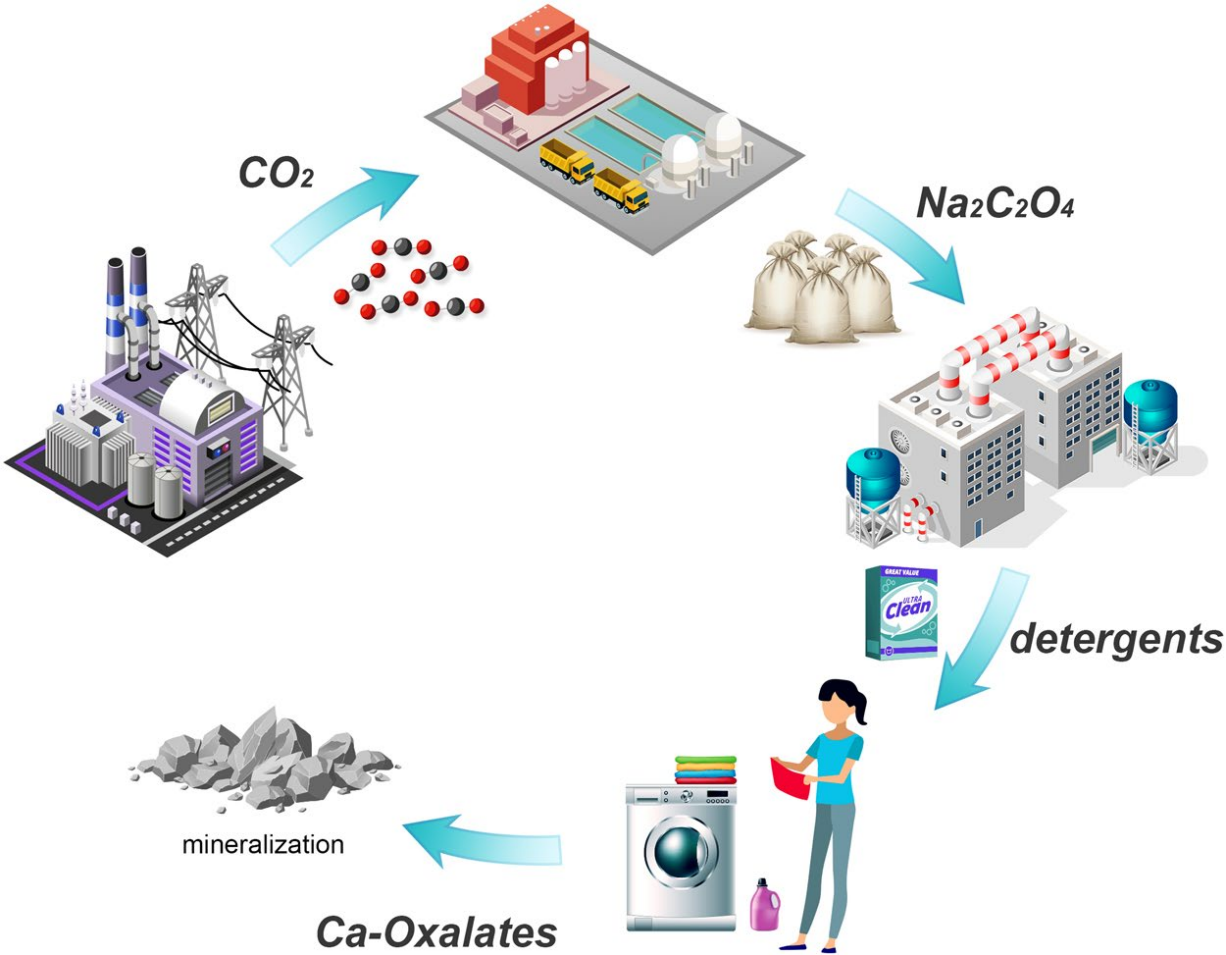
The whole process of the capture, utilization and mineralization of CO_2_.

On the basis of the technologies described in references, we introduce a strategy that integrates CO_2_ capture, utilization, and mineralization into one system (Fig. 5). In the capture process, CO_2_ is absorbed by an inorganic base and then used to produce sodium oxalate; thus, enormous energy consumption to regenerate the absorbent can be avoided.

As can be seen in the experimental results, sodium oxalate has good performance in calcium removal and detergency. The obtained calcium oxalates have good water and thermal stability. In summary, it is a feasible way of realizing carbon mineralization using sodium oxalate as a detergent builder. As a commodity product, safety of detergent ingredient is an issue people concerned. Through the acute oral toxicity data (shown in Table S7), we think the toxicity of sodium oxalate will not limit its use in detergent.

This strategy has many advantages. Sodium oxalate is prepared from CO_2_, and the manufacturing process is environment friendly. The actual carbon mineralization is realized in the laundry process, so no extra equipment and energy is required. Doing laundry has been a habit for thousands of years and is an indispensable part of peoples’ lives. Thus, people’s daily cleaning activities can help to reduce carbon dioxide emissions while hardly increasing energy consumption. On the basis of economic and environmental considerations, this strategy will result in a reduction in the emission of fossil-fuel-derived carbon dioxide, which is beneficial to the environment.

Overall, this work is valuable and provides a potential available route in industry. However, some problems need further investigation, for example more detailed work needed to evaluate the footprint of the C_2_O_4_^2−^ in the environment after washing process and its environmental effects.

## Methods

### Raw chemicals

CaCl_2_ and MgCl_2_·6H_2_O were analytical reagents purchased from Accelerating Scientific and Industrial Development. Sodium oxalate, STPP, Na_2_CO_3_, and EDTA of analytical grade, SDBS with active matter content of 95%, and sodium carboxymethyl cellulose (CMC-Na) with viscosity of 300–800 mPa·s were purchased from Shanghai Aladdin Bio-Chem Technology Co., Ltd. China Research Institute of Daily Chemical Industry supplied 85% MES, 70% AES, and 99% AEO_9_. Na_2_SO_4_ was purchased from Sinopharm Chemical Reagent Co., Ltd. Sodium silicate (Qingdao Paohua Jian Co., Ltd.) and zeolite 4A (Nafine Chemical Industry Group Co., Ltd.) were of industrial grade.

### Material preparation

#### Builder samples

STPP and sodium oxalate were carefully dried for 2 h at 110 °C in a laboratory oven and cooled to room temperature in a desiccator. Zeolite 4A was stored in a humidistat (a closed container with saturated NH_4_Cl solution).

#### Standard CaCl_2_ solution

The right quantity of CaCl_2_ was weighed to prepare 0.05 mol/L CaCl_2_ solution. Ethylenediaminetetraacetic acid disodium salt (EDTA) and eriochrome black T indicator were used to calibrate the concentration. The CaCl_2_ solution was then diluted with water at ratios of 1:10 or 1:20.

#### Soiled fabrics

The soiled fabrics (purchased from China Research Institute of Daily Chemical Industry) were cut into 60 × 60 mm pieces. A group of soiled fabrics contained four pieces with JB-01, four pieces with JB-02, and four pieces with JB-03.

#### Hard water

CaCl_2_ (1.670 g) and MgCl_2_·6H_2_O (2.037 g) were dissolved in distilled water and made into 10 L to prepare hard water.

### Calcium-removing property

The CRC was measured by complexometric titration as reported in many articles^31,54^. A 5 mmol/L standard solution of CaCl_2_ was prepared, and its pH was adjusted to between 10 and 10.5 with 2.5 mol/L NaOH solution. This solution (500 mL) was transferred into a 1000 mL round-bottom beaker and heated to a certain temperature in a constant-temperature water bath (10, 30, or 50 °C). The precisely weighed sample was poured into the beaker and agitated for 20 min at 500 rpm. The mixture was then filtered using slow qualitative filter paper. The filtrates (50 mL) were subjected to complexometric titration against 0.01 mol/L EDTA standard solution with eriochrome black T indicator, which produced a sharp color change from wine red to pure blue. Data are the average of the three separate measurements. The CRC was calculated as mass ratio using Eq. 1:

1 $${\rm{CRC}}\,({\rm{mg}}\,{{\rm{CaCO}}}_{3}/g)=\frac{{\rm{100.08}}\times ({\rm{500}}{C}_{0}-10{C}_{1}{V}_{1})}{m}$$

where 100.08 is the molar mass of calcium carbonate (CaCO_3_); *C*_0_ is the molar concentration of CaCl_2_ standard solution, in mol/L; V_1_ is the volume of EDTA standard solution consumed in the titration, in mL; *C*_1_ is the molar concentration of EDTA standard solution consumed in the titration, in mol/L; and *m* is the mass of the dry builder sample taken for the test, in g.

In the CRR experiment, the 2.5 mmol/L CaCl_2_ solution in demineralized water was prepared, and its pH was adjusted to 10–10.5. The Ca ISE (LeiCi, PCa-1-01) and calomel electrode used as the reference were immersed in the CaCl_2_ solution and heated in a water bath with constant agitation by a magnetic stirrer. When the temperature was increased to the specified temperature (10, 30, and 50 °C), the builder was poured into the solution and timed with a stopwatch; the concentration of Ca^2+^ was noted at certain times. These measurements were repeated three times, and the mean was taken to eradicate any discrepancies. The rate curves were drawn with time as abscissa and Ca^2+^ concentration as ordinate. Data are the average of the two separate measurements and the results of the measurements agree with a deviation of 2%.

### Detergency performance evaluation

The detergency performance of sodium oxalate as a detergent builder was evaluated in a vertical cleaner (RHLQ-III, China Research Institute of Daily Chemical Industry). The whole washing process consisted of main washing and rinse washing. In the main wash, a group of soiled fabrics were washed in a beaker with 1 L of hard water. The operating parameters used in the present work include detergent concentration (0.2%), agitating speed (120 rpm), washing time (20, 40, and 60 min), and washing temperature (10, 30, and 50 °C). In the rinse wash, the washed fabrics were rinsed twice with tap water for 2 min and manually dried for 30 s. The fabrics were dried at room temperature. The reflectance of pre-washed and post-washed fabrics was measured by an SC-80 colorimeter (Beijing Kangguang Optical Instrument Co., Ltd.). The detersive power was calculated through Eq. 2:

2 $${\rm{R}}=\frac{({F}_{2}-{F}_{1})}{{\rm{n}}}$$

where *F*_1_ is the average reflectance of pre-washed soiled fabrics (%), *F*_2_ is the average reflectance of post-washed soiled fabrics (%), n is quantity of the soiled fabrics and *R* is the detersive power value of the testing sample detergent (%). The relative detersive ratio value of the detergent is *P* = *R*/*R*_0_, where *R*_0_ is the detersive power value of normal detergent (%).

### Characterization of collected solids

The detergent used in this part is formulated as follows (in wt.%): anionic surfactant (16.0), AEO_9_ (4.0), sodium oxalate (20.0), sodium carbonate (10.0), sodium silicate (6.0), carboxymethyl cellulose (2.0), and sodium sulfate (up to 100).

The different suspensions were filtered through membrane filters (0.45 μm Millipore), and the solids were collected and dried at room temperature. Powder XRD patterns were collected on an X-ray diffractometer (MiniFlexII, Rigaku) using Cu Kα radiation (λ = 1.5418 Å). For the SEM studies, all samples were coated with gold prior to examination by a Hitachi TM-3000 with a field emission source and operated at an accelerating voltage of 15 kV.

The thermal stability of solid samples was analyzed at a heating rate of 2 °C/min and in a temperature range of room temperature to 1000 °C in air atmosphere. The COM and COD used in washing experiments were prepared according to the literature and carefully dried at 110 °C. Subsequently, 1.000 g of precisely weighed COM and COD was transferred to a dry filter and washed with 5.0 L of municipal water, dried at 110 °C, and weighed again to calculate the solid retention. The COM and COD were characterized by XRD and SEM.

3 $${\rm{solid}}\,{\rm{retention}}=\frac{{m}_{1}}{{m}_{0}}$$

where *m*_0_ is the solid mass before washing and *m*_1_ is the solid mass after washing. Data are the average of the three separate measurements.

## Supplementary information

Supplementary Information

## Data Availability

All the data supporting the findings are available from the corresponding author upon reasonable request.
