# Supplementary material for: A New Route for Indirect Mineralization of Carbon Dioxide–Sodium Oxalate as a Detergent Builder
Source: Sci Rep. 2019 Sep 6;9:12852. doi: 10.1038/s41598-019-49127-8 (PMC6731234; doi:10.1038/s41598-019-49127-8)
Supplement: Supplementary file 1 — Supplementary Information [file 41598_2019_49127_MOESM1_ESM.docx]

A New Route for Indirect Mineralization of Carbon Dioxide–Sodium Oxalate as a Detergent Builder

Chen Li, Lijie Wang, Min Yuan, Hong Xu* and Jinxiang Dong*

^1^Research Institute of Special Chemicals, College of Chemistry and Chemical Engineering, Taiyuan University of Technology, Taiyuan 030024, Shanxi, P.R. China.

Supporting information

##
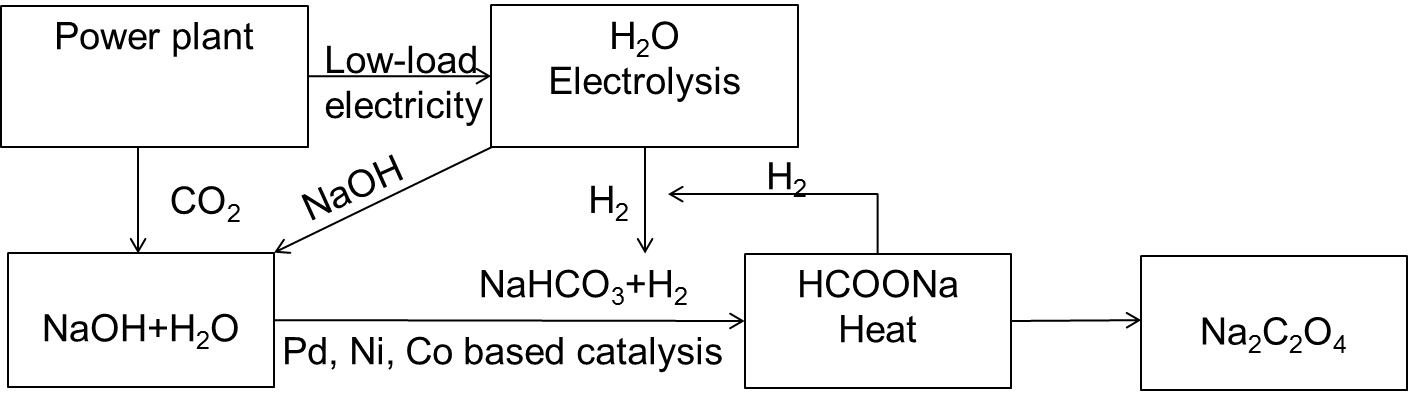


**Figure S1.** The detail of the production process for Na_2_C_2_O_4_

In China, coal-fired power is the main source of electricity. A typical flue gas of coal-fired power contains 12 - 15% CO_2_ (Carbon Capture, New York: Springer Sciences & Business Media, 2012: p6). Capture and enrichment of the low concentrate CO_2_ will need high energy consumption.

Here, we propose a novel CO_2_ utilization idea to directly use the lower concentration CO_2_ from power plants to produce chemicals.

First, the CO_2_ is absorbed by NaOH solution to produce NaHCO_3_. This is a mature technology. CO_2_ captured by NaOH solution is a low energy consumption process.

Second, reacting NaHCO_3_ with H_2_ can obtained HCOONa, which has been reported in many articles (Dmitri A et al., Catalysis Reviews, 2018, 60(4): 566-593; *ChemComm,* 1999, 971-972;). The captured CO_2_ does not need desorption process, which can avoid a lot of energy. This reaction can be carried out by Pd, Ni and Co-based etc. as catalyst.

In this production technology, hydrogen is obtained by two routes. One is the conversion of HCOONa to Na_2_C_2_O_4_ by heating method. The other is water electrolysis by using the off-peak electrical energy. According to reports, when coal-fired power unit run in low-load operation, problems of high energy-consumption, environmental pollutants and safety barriers have always troubled coal-fired power plants. Therefore, the big peak-valley difference and the limited peaking regulation lead to the waste of electrical energy. (*Energy and Buildings*, 2010, 9:1552-1560; *Applied Energy*, 185:872-884)

Third, the HCOONa is heated to obtain Na_2_C_2_O_4_, which is used in the existing process to produce Na_2_C_2_O_4_. Typically, the temperature is from 400-440 °C, preferably 400-420 °C (*Journal of Beijing University of Chemical Technology,* 2007, 34(6):566-569).

Table S1. Formulations in the detergency performance evaluation

| Ingredient | Percent/% |
| --- | --- |
| Anion surfactant^[a]^ | 16 |
| AEO9 | 4 |
| Builder^[b]^ | 20/25/30 |
| Sodium carbonate | 10 |
| Sodium Silicate | 6 |
| CMC-Na | 2 |
| Sodium sulfate | Up to 100 |

[a] is one of SDBS, MES and AES,the content [b] Builder is one of sodium oxalate, STPP and zeolite 4A

## Table S2. The normal detergent formulation according to GB/T 13174-2008

| Ingredient | Percent/% |
| --- | --- |
| SDBS | 16 |
| STPP | 4 |
| Na_2_SiO_3_ | 30 |
| Na_2_CO_3_ | 10 |
| CMC | 6 |
| Na_2_SO_4_ | 2 |

[1]. Chinese National Standard, GB/T 13174-2008: Deternination of detergency and cycle of washing property for laundry detergents, 2008

## Table S3. Relative detersive ratio values of different detergent formulations with different builder contents

|  |  | SDBS | | | MES | | | AES | | |
| --- | --- | --- | --- | --- | --- | --- | --- | --- | --- | --- |
|  | Builder  Content（%） | 20 | 25 | 30 | 20 | 25 | 30 | 20 | 25 | 30 |
| Sodium oxalate | JB-01 | 0.99 | 1.02 | 1.06 | 1.02 | 1.02 | 1.02 | 1.06 | 1.06 | 1.10 |
|  | JB-02 | 0.92 | 1.06 | 1.32 | 1.25 | 1.35 | 1.44 | 1.23 | 1.45 | 1.55 |
|  | JB-03 | 1.05 | 1.10 | 1.24 | 1.10 | 1.15 | 1.16 | 1.24 | 1.34 | 1.50 |
| STPP | JB-01 | 0.98 | 1.01 | 1.06 | 1.00 | 1.00 | 1.08 | 1.02 | 1.02 | 1.04 |
|  | JB-02 | 1.12 | 1.11 | 1.13 | 0.88 | 0.97 | 1.18 | 0.95 | 1.08 | 1.15 |
|  | JB-03 | 1.12 | 1.20 | 1.28 | 1.03 | 1.10 | 1.14 | 1.35 | 1.53 | 1.63 |
| Zeolite 4A | JB-01 | 0.93 | 0.96 | 0.94 | 0.94 | 0.91 | 0.90 | 0.98 | 1.00 | 1.03 |
|  | JB-02 | 0.81 | 0.79 | 0.79 | 0.84 | 0.85 | 0.83 | 1.13 | 1.16 | 1.20 |
|  | JB-03 | 0.82 | 0.85 | 0.87 | 0.94 | 0.96 | 1.04 | 1.13 | 1.18 | 1.20 |

The relative detergency of each detergent composition was calculated on the basis of the detergency obtained from the test with normal detergent at 30^o^C, 20 min as 1.00.

Soils: JB-01 carbon black soil fabric ;JB-02 mixed protein soil fabric and JB-03 artificial sebum fabric.

## Table S4. Relative detersive ratio values of different detergent formulations with different times when the builder content is 25%, washing at 30 °C

|  |  | SDBS | | MES | | AES | |
| --- | --- | --- | --- | --- | --- | --- | --- |
|  | Washing  time (min) | 60 | 40 | 60 | 40 | 60 | 40 |
| Sodium oxalate | JB-01 | 1.28 | 1.19 | 1.25 | 1.20 | 1.11 | 1.07 |
|  | JB-02 | 1.80 | 1.34 | 2.00 | 1.80 | 1.76 | 1.56 |
|  | JB-03 | 1.86 | 1.56 | 2.32 | 1.68 | 2.34 | 2.09 |
| STPP | JB-01 | 1.29 | 1.16 | 1.11 | 1.20 | 1.16 | 1.13 |
|  | JB-02 | 1.70 | 1.29 | 1.44 | 1.38 | 1.41 | 1.24 |
|  | JB-03 | 1.99 | 1.71 | 1.89 | 1.46 | 1.84 | 1.62 |
| Zeolite 4A | JB-01 | 1.16 | 1.07 | 1.20 | 1.14 | 1.07 | 1.02 |
|  | JB-02 | 1.58 | 1.28 | 1.57 | 1.22 | 1.78 | 1.53 |
|  | JB-03 | 1.58 | 1.35 | 1.97 | 1.38 | 1.91 | 1.66 |

The relative detergency of each detergent composition was calculated on the basis of the detergency obtained from the test with normal detergent at 30^o^C, 20 min as 1.00.

## Table S5. Relative detersive ratio values of different detergent formulations with different temperature washing for 20 min

|  |  | SDBS | | MES | | AES | |
| --- | --- | --- | --- | --- | --- | --- | --- |
|  | Temperature (°C) | 10 | 50 | 10 | 50 | 10 | 50 |
| Sodium oxalate | JB-01 | 1.00 | 1.36 | 0.96 | 1.27 | 0.94 | 1.21 |
|  | JB-02 | 1.02 | 1.61 | 0.87 | 1.76 | 1.03 | 1.78 |
|  | JB-03 | 0.87 | 1.42 | 0.97 | 1.69 | 1.26 | 1.93 |
| STPP | JB-01 | 0.97 | 1.40 | 0.96 | 1.27 | 0.91 | 1.16 |
|  | JB-02 | 0.88 | 1.27 | 0.86 | 1.30 | 1.04 | 1.20 |
|  | JB-03 | 1.14 | 1.67 | 0.67 | 1.69 | 0.92 | 1.64 |
| Zeolite 4A | JB-01 | 0.94 | 1.32 | 0.95 | 1.18 | 0.95 | 1.02 |
|  | JB-02 | 0.91 | 1.61 | 0.83 | 1.66 | 0.92 | 1.57 |
|  | JB-03 | 0.73 | 1.20 | 0.69 | 1.46 | 0.93 | 1.61 |

The relative detergency of each detergent composition was calculated on the basis of the detergency obtained from the test with normal detergent at 30^o^C, 20 min as 1.00.


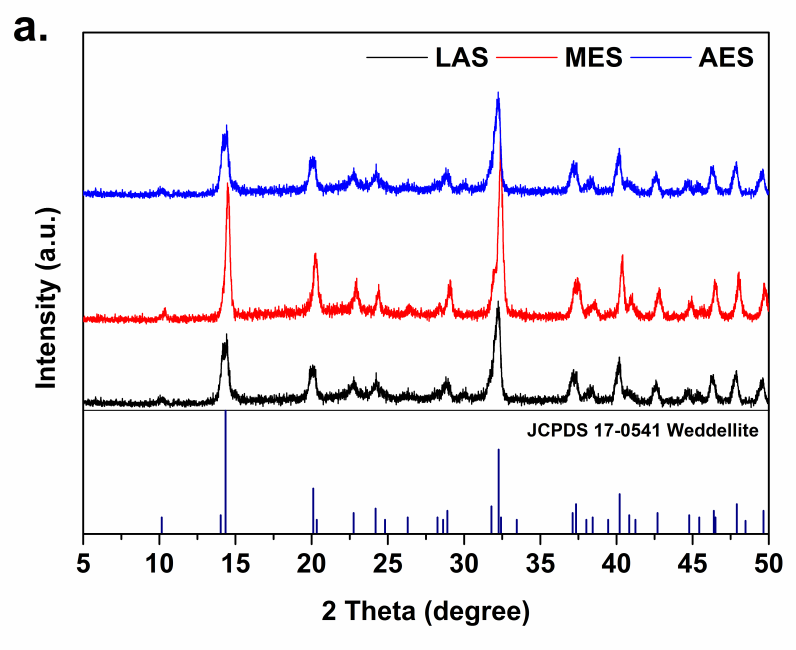

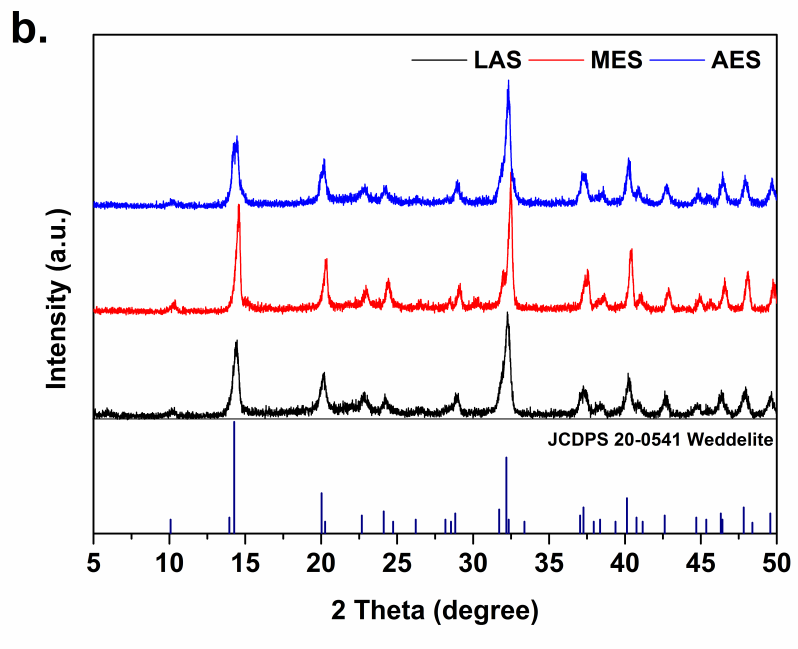

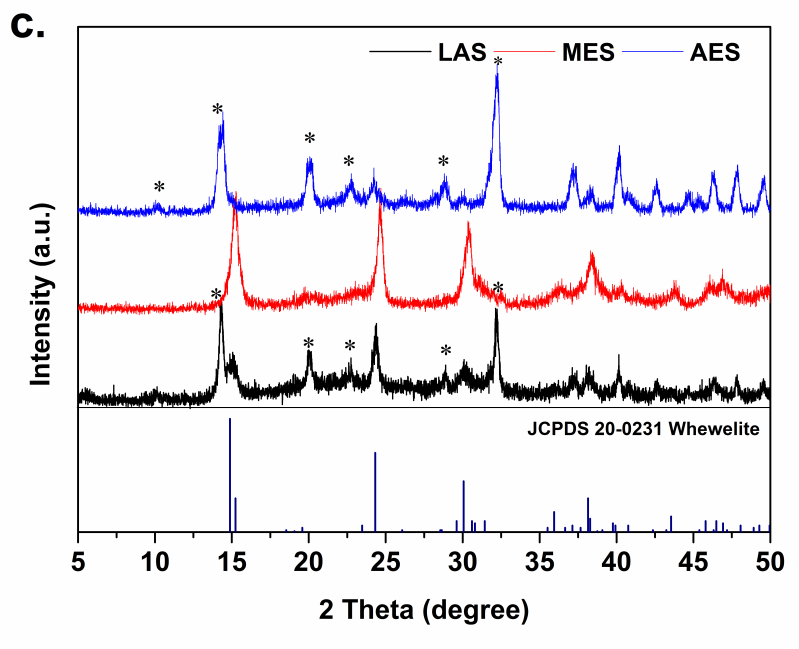


**Figure S2**. XRD patterns of the solid samples collected from CRC experiments at different temperatures





## Figure S3. XRD patterns of the solid samples collected from CRC experiments at different temperatures


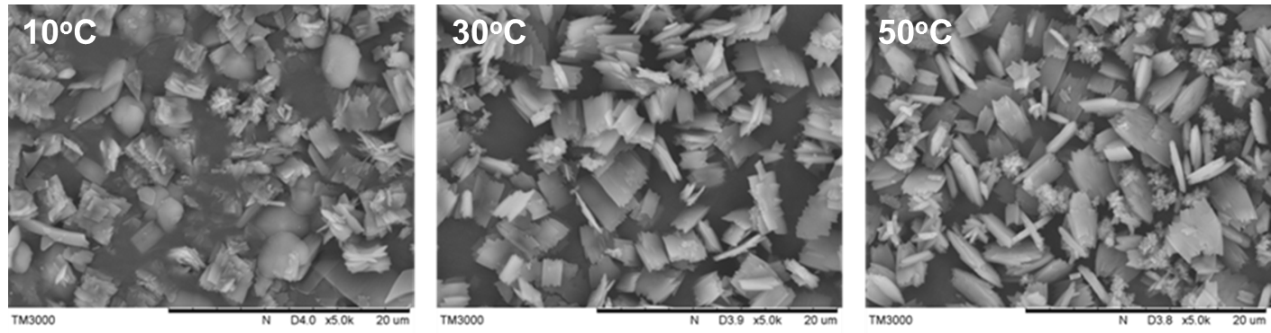


## Figure S4. SEM images of the solid samples collected from CRC experiments at different temperatures: 10^o^C (left), 30^o^C (middle) and 50^o^C (right)

## Table S6. Formulations used to collect solids at different conditions

|  | 10 °C | | | 30 °C | | | 50 °C | | |
| --- | --- | --- | --- | --- | --- | --- | --- | --- | --- |
| Formulation number | 1 | 2 | 3 | 4 | 5 | 6 | 7 | 8 | 9 |
| SDBS | 16 | 0 | 0 | 16 | 0 | 0 | 16 | 0 | 0 |
| MES | 0 | 16 | 0 | 0 | 16 | 0 | 0 | 16 | 0 |
| AES | 0 | 0 | 16 | 0 | 0 | 16 | 0 | 0 | 16 |
| AEO-9 | 4 | 4 | 4 | 4 | 4 | 4 | 4 | 4 | 4 |
| Na_2_C_2_O_4_ | 25 | 25 | 25 | 25 | 25 | 25 | 25 | 25 | 25 |
| Na_2_SiO_3_ | 6 | 6 | 6 | 6 | 6 | 6 | 6 | 6 | 6 |
| Na_2_CO_3_ | 10 | 10 | 10 | 10 | 10 | 10 | 10 | 10 | 10 |
| CMC | 2 | 2 | 2 | 2 | 2 | 2 | 2 | 2 | 2 |
| Na_2_SO_4_ | 37 | 37 | 37 | 37 | 37 | 37 | 37 | 37 | 37 |

The numbers in the table is the present of each chemistry in the formulation.

**

**

## Figure S5. XRD patterns of solid collected from water solutions without washing at different conditions (Supplementary Table 5)

**
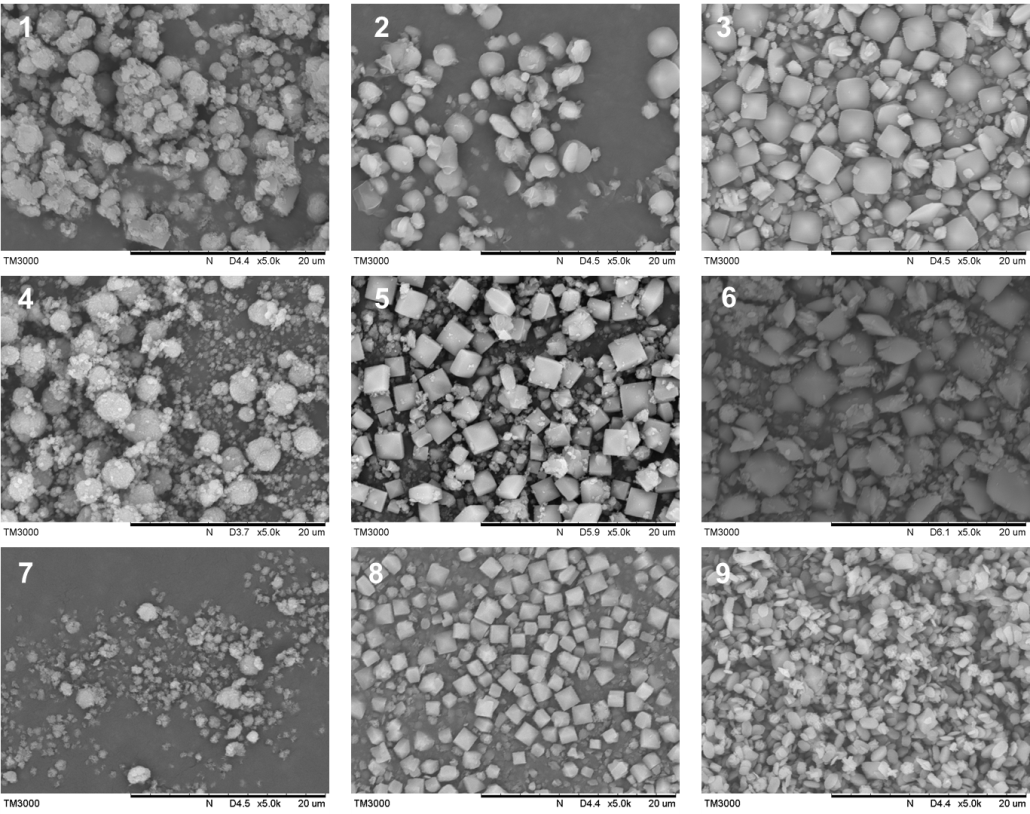
**

## Figure S6. SEM images of solid collected from waters without washing at different conditions (Supplementary Table 6)





## Figure S7. TG plots for samples from different conditions (Supplementary Table 6)


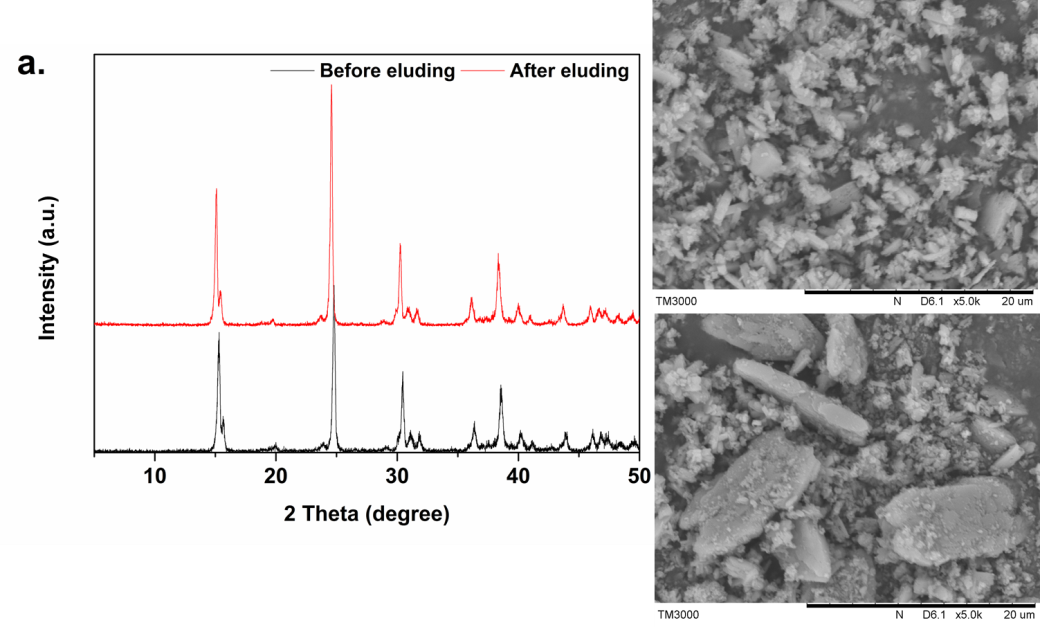


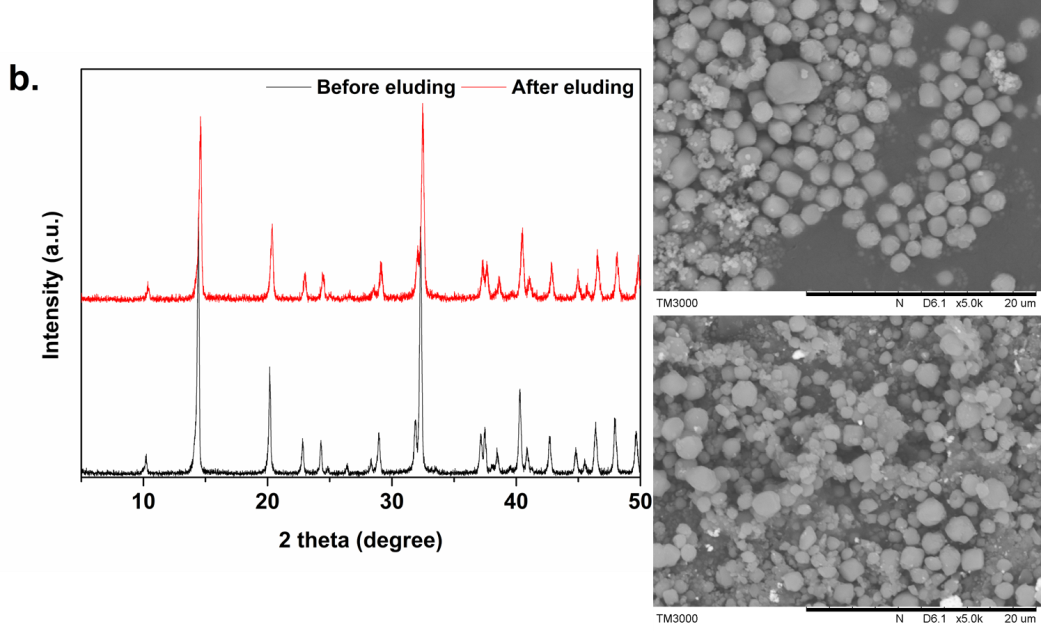


## Figure S8. XRD patterns and SEM images of calcium oxalate before and after eluding a.COM b.COD

## Table S7. Acute oral toxicity of common detergent ingredients in rats

|  | LD_50_(g/kg body weight) |
| --- | --- |
| SDBS | 1.3-2.5 ^1^ |
| C14-MES | 0.5-1.0 ^2^ |
| AE3S | 1.2-1.6 ^3^ |
| Sodium oxalate | 11.16 ^4^ |

## Michael W R. Metabolism of linear alkylate sulfonate and alkyl benzene sulfonate in albino rats. *Toxicology and applied pharmacology*, 12(3): 473-485 (1968).

## Sato, Jun, et al. Toxicity studies of tetradecanoic acid, 2-sulfo-, 1-methylester, sodium salt (C14-MES). *The Journal of toxicological sciences*,30(4): 339-347 (2005).

1. Walker A. I. T. et al. Toxicity of sodium lauryl sulphate, sodium lauryl ethoxysulphate and corresponding surfactants derived from synthetic alcohols. *Fd. Cosmet. Toxicol.* 5, 763-769 (1967)
2. Safety Data Sheet from Sigma-Aldrich website
